# Impact of age on the survival of patients with liver cancer: an analysis of 27,255 patients in the SEER database

**DOI:** 10.18632/oncotarget.2719

**Published:** 2015-01-21

**Authors:** Wenjie Zhang, Beicheng Sun

**Affiliations:** ^1^ Liver Transplantation Center of the First Affiliated Hospital and State Key Laboratory of Reproductive Medicine, Nanjing Medical University, Nanjing, Jiangsu Province, P.R. China

**Keywords:** age, liver cancer, SEER

## Abstract

**Background & Aims:**

The risk of liver cancer (LC) is regarded as age dependent. However, the influence of age on its prognosis is controversial. The aim of our study was to compare the long-term survival of younger versus older patients with LC.

**Methods:**

In this retrospective study, we searched Surveillance, Epidemiology, and End-Results (SEER) population-based data and identified 27,255 patients diagnosed with LC between 1988 and 2003. These patients were categorized into younger (45 years and under) and older age (over 45 years of age) groups. Five-year cancer specific survival data was obtained. Kaplan–Meier methods and multivariable Cox regression models were used to analyze long-term survival outcomes and risk factors.

**Results:**

There were significant differences between groups with regards to pathologic grading, histologic type, stage, and tumor size (*p* < 0.001). The 5-year liver cancer specific survival (LCSS) rates in the younger and older age groups were 14.5% and 8.4%, respectively (*p* < 0.001 by univariate and multivariate analysis). A stratified analysis of age on cancer survival showed only localized and regional stages to be validated as independent predictors, but not for advanced stages.

**Conclusions:**

Compared to older patients, younger patients with LC have a higher LCSS after surgery, despite the poorer biological behavior of this carcinoma.

## INTRODUCTION

Liver cancer (LC) is the fifth most common malignant cancer in men and the seventh in women, and is ranked as the third leading cause of cancer-related deaths globally [1]. Primary liver cancer can usually be classified histologically as hepatocellular carcinoma (HCC), intrahepatic cholangiocarcinoma (ICC), or combined HCC and ICC [2]. This malignancy exhibits a remarkable gender disparity in male patients [3]. Over the last few years, accumulating evidences has suggested that the incidence of LC continues to increase [4]. In 2013, an estimated 30,640 adults (22,720 men and 7920 women) in the United States were diagnosed with primary liver cancer [5]. The incidence of LC is also increasing rapidly in Asian countries and Asians have been affected twice as much than Africans [6]. Generally, LC is considered as a malignancy influencing mainly those aged 65 and older, with 74% of cases occurring in men [7].

Age has a prognostic implication in many solid cancers. Chen et al. reported that age may play a paradoxical role on the prognosis of HCC [8]. Young patients with LC are considered to have a poorer prognosis, since they exhibit an advanced tumor stage and poor pathologic grading [9, 10]. However, some studies have argued that while young LC patients have unfavorable clinicopathologic characteristics, they have a better long-term survival than elderly patients [11, 12]. These varying results on LC in young patients may due to limited sample sizes or single-institution experiences. To further clarify the issue of age on LC prognosis, Surveillance, Epidemiology, and End Results (SEER) population-based data were analyzed in our study.

## RESULTS

### Clinicopathologic parameters of patients

Of 27,255 LCs diagnosed during the 15-year study period (between 1988 and 2003) in the SEER database, 19324 (70.9%) were males and 7931 (29.1%) were females. The median age was 39 in the younger age group and 66 in the older age group. The median follow-up period was 17 months. Patient demographics and pathologic features are summarized in Table 1.

**Table 1:** Characteristics of Patients from SEER Database by age

Characteristic	Total	Young Group	Elderly Group	*p* value
	27255	2102	25153	
Media follow up (mo)		24	16	*P* < 0.001
(IQR)		1–20	1–14	
Years of diagnosis				*P* < 0.001
1988–1993	4505	391	4114	
1994–1999	8525	698	7827	
2000–2003	14225	1003	13222	
Sex				*P* < 0.001
Male	19324	1582	17742	
Female	7931	520	7411	
Race				*P* < 0.001
Caucasian	17891	1109	16782	
African American	2998	277	2721	
Others	6287	708	5579	
Unknowns	79	8	71	
Primary site				*P* < 0.001
Liver	24317	1924	22393	
Intrahepatic bile duct	2938	178	2760	
Pathological grading				*P* < 0.001
High/Moderate	6548	481	6067	
Poor/undifferentiation	3023	317	2706	
Unknown	17684	1304	16380	
Histological Type				*P* < 0.001
Hepatocellular carcinoma	23547	1846	21701	
Cholangiocarcinoma	3502	228	3274	
Combined	206	28	178	
Stage				*P* < 0.001
Localized	9100	627	8473	
Regional	7077	627	6450	
Distant	5467	541	4926	
Unstaged	5611	307	5304	
Tumor size				*P* < 0.001
< 3cm	2088	169	1919	
3–5cm	4155	255	3900	
> 5cm	7618	697	6921	
Not stated	13394	981	12413	

### Clinicopathologic differences between groups

As illustrated in Table 1, there were significant differences observed between the two groups. Compared with the older age group, the younger age group demonstrated differences with regards to the calendar years of diagnosis (more frequent in 2000–2003, *p* < 0.001), gender (more frequent in females, *p* < 0.001), race (less frequent in Caucasians, *p* < 0.001), primary site (more frequent in the intrahepatic bile duct, *p* < 0.001), pathologic grade (less high/moderate in grade, *p* < 0.001), histologic type (less hepatocellular carcinoma, *p* < 0.001), stage (less localized, *p* < 0.001), and tumor size (< 3 cm, *p* < 0.001). After further analyzing these differences in HCC and ICC, respectively, as shown in Table S1A and Table S1B, these differences were also observed in HCC patients. However, in ICC patients only stage showed a significant difference.

### Impact of age on LC survival outcomes

**Figure 1:**
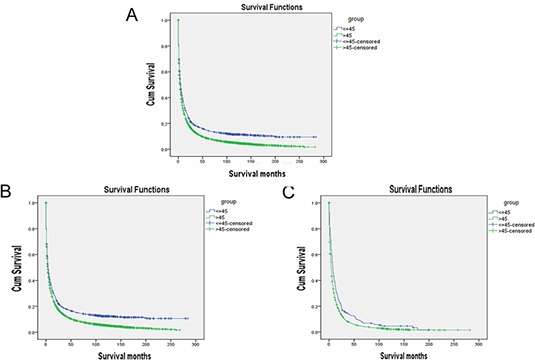
Survival curves in patients according to age status LC patients: Young group vs. Elderly group, χ2 = 85.221, *P* < 0.001, HCC patients: Young group vs. Elderly group, χ2 = 69.408, *P* < 0.001, ICC patients: Young group vs. Elderly group, χ2 = 16.883, *P* < 0.001

The univariate log-rank test showed that the overall 5-year liver cancer specific survival (LCSS) was 14.5% and 8.4% in the younger and older age groups, respectively ( *p* < 0.001) (Figure 1A). Stratified analysis of histologic type (HCC and ICC) confirmed these differences (Figure 1B and 1C). Moreover, male ( *p* < 0.05), gender, an early year of diagnosis (1988–1993), African-American race, intrahepatic bile duct, poor/undifferentiated grade, cholangiocarcinoma or combined hepatocellular and cholangiocarcinomas, higher stage, and larger tumor size ( *p* < 0.001), were regarded as significant risk factors for a poorer prognosis by univariate analysis (Table 2). Multivariate analysis was also performed by the Cox regression model. The following seven factors were found to be independent prognostic factors (Table 3), including year of diagnosis (1994–1999, hazard ratio (HR) 1.063, 95% confidence interval (CI) 0.958–1.181; 2000–2003, HR 0.900, 95% CI 0.816–0.992), age (> 45, HR 1.286, 95% CI 1.158–1.427), gender (female, HR 0.887, 95% CI 1.158–1.427), race (African-American, HR 1.150, 95% CI 1.040–1.270; others, HR 0.859, 95% CI 0.801–0.922), pathologic grading (poor/undifferentiated, HR 1.431, 95% CI 1.342–1.526), stage (regional, HR 1.540, 95% CI 1.444–1.643; distant, HR 2.562, 95% CI 2.348–2.796), tumor size (3–5 cm, HR 1.524, 95% CI 1.387–1.673; > 5 cm, HR 1.932, 95% CI 1.768–2.110). However, no statistical difference were observed with regards to primary site ( *p* = 0.584) and histologic type ( *p* = 0.387) according to multivariate survival analysis. Meanwhile, age, pathological grading, and stage were also identified as independent prognostic factors when the analysis was performed separately by histologic type (Table S2 and S3).

**Table 2:** Univariate survival analyses of LC patients according to various clinicopathological variables

Variable	n	5-year LCSS (%)	Log rank χ2 test	*p* value
Years of diagnosis			454.61	*P* < 0.001
1988–1993	4505	4.3%		
1994–1999	8525	7.4%		
2000–2003	14225	11.2%		
Sex			4.69	*P* < 0.05
Male	19324	8.8%		
Female	7931	9.2%		
Age			85.22	*P* < 0.001
≤ 45	2102	14.5%		
> 45	25153	8.4%		
Race			199.66	*P* < 0.001
Caucasian	17891	8.5%		
African American	2998	5.7%		
Others*	6366	11.5%		
Primary site			23.04	*P* < 0.001
Liver	24317	9.4%		
Intrahepatic bile duct	2938	4.6%		
Pathological grading			597.76	*P* < 0.001
High/Moderate	6548	17.8%		
Poor/undifferentiation	3023	7.0%		
Histological Type			20.99	*P* < 0.001
Hepatocellular carcinoma	23547	9.5%		
Cholangiocarcinoma	3502	5.1%		
Combined	206	6.8%		
Stage			3135.26	*P* < 0.001
Localized	9100	18.5%		
Regional	7077	5.8%		
Distant	5467	1.5%		
Tumor size			953.15	*P* < 0.001
< 3cm	2088	31.3%		
3–5cm	4155	15.7%		
> 5cm	7618	8.2%		

* including other (American Indian/AK Native, Asian/Pacific Islander) and unknowns.

**Table 3:** Multivariate Cox model analyses of prognostic factors of LC

Variable	Hazard Ratio	95%CI	*p* value
Years of diagnosis			*P* < 0.001
1988–1993	1		
1994–1999	1.063	0.958–1.181	
2000–2003	0.900	0.816–0.992	
Sex			*P* < 0.001
Male	1		
Female	0.887	0.830–0.948	
Age			*P* < 0.001
≤ 45	1		
> 45	1.286	1.158–1.427	
Race			*P* < 0.001
Caucasian	1		
African American	1.150	1.040–1.270	
Others*	0.859	0.801–0.922	
Primary site			0.584
Liver	1		
Intrahepatic bile duct	1.053	0.875–1.269	
Pathological grading			*P* < 0.001
High/Moderate	1		
Poor/undifferentiation	1.431	1.342–1.526	
Histological Type			0.387
Hepatocellular carcinoma	1		
Cholangiocarcinoma	0.907	0.772–1.065	
Combined	1.094	0.827–1.448	
Stage			*P* < 0.001
Localized	1		
Regional	1.540	1.444–1.643	
Distant	2.562	2.348–2.796	
Tumor size			*P* < 0.001
< 3cm	1		
3–5cm	1.524	1.387–1.673	
> 5cm	1.932	1.768–2.110	

* including other (American Indian/AK Native, Asian/Pacific Islander) and unknowns.

### Association of age and cancer survival on different stages: a stratified analysis

We further analyzed whether age was associated with 5-year LCSS in different stages. The univariate analysis of age on LCSS showed that younger patients had an increased 5-year LCSS across several subgroups (Table 4). Multivariate Cox regression analyses were performed for different stages; age was validated as an independent predictor of survival in the localized stages (elderly, HR 1.514, 95% CI 1.292–1.774, *p* < 0.001) and regional stage (elderly, HR 1.262, 95% CI 1.058–1.505, *p* < 0.001), but not in distant stages (*P* = 0.302) (Table 5).

**Table 4:** Univariate analysis of Age on LCSS based on different stages

Variable	n	5-year LCSS (%)	Log rank χ2 test	*p* value
Localized				
Age			107.987	*P* < 0.001
≤ 45	627	33.2%		
> 45	8473	17.4%		
Regional				
Age			13.164	*P* < 0.001
≤ 45	627	8.5%		
> 45	6450	5.5%		
Distant				
Age			21.172	*P* < 0.001
≤ 45	541	2.5%		
> 45	4926	1.4%		

**Table 5:** Multivariate Cox model analyses of prognostic factors of LC on different stages

Variable	Hazard Ratio	95%CI	*p* value
Localized			
Age			*P* < 0.001
≤ 45	1		
> 45	1.514	1.292–1.774	
Regional			
Age			*P* < 0.05
≤ 45	1		
> 45	1.262	1.058–1.505	
Distant			
Age			0.302
≤ 45	1		
> 45	0.881	0.693–1.120	

*P* values were adjusted for years of diagnosis, sex, age, race, primary site, pathological grading, histological type, stage, tumor size as covariates between the two groups.

## DISCUSSION

Whether age affects the prognosis of LC is controversial, as well as the definition of young patients. The conflicting data may result from the heterogeneity among these studies. Some studies used 50 years as the cutoff age [13, 14], while other studies used 40, 30, or 45 years [15–18]. It is difficult to compare younger and older age groups due to lack of a unified standard definition. Since the morbidity of LC is relatively rare and stable until 45 years [19], which was consistent with our results (8.68% in years of 1988–1993, 8.19% in years of 1994–1999, 7.05% in years of 1994–1999), we defined 45 years as the cutoff for younger age, as most studies reported.

The age-adjusted rates of LC increased two-fold from the mid-1980s to the late 1990s. As incidence rates increased, the distribution of LC has shifted from elderly patients toward relatively younger ones [6]. Young patients with gastric and breast cancer have a poorer prognosis than elderly ones [20, 21]. Conversely, young colorectal and thyroid cancer patients have a better long-term survival [22, 23]. Various studies have also reported that age plays a paradoxical role on the prognosis of HCC [8]. Cho et al. demonstrated that young patients had poorer survival rates than elderly patients [24]. This reduction in survival resulted from a more advanced tumor stage at diagnosis, despite the fact that they had better liver function. These results were confirmed by Shimada et al. [25]. In addition, young patients tended to exhibit larger tumor sizes and poorer differentiation compared with older ones [26]. Yang et al. found that women under 55 years had a superior survival to men, which confirmed the protective role of estrogens [27]. However, our study showed that, regardless of sex, the younger group had a better 5-year LCSS than the older age group (14.5% and 8.4%, respectively). As younger patients might have poorer biological behavior, this may be compensated by better liver function, which contributes to longer survival. Moreover, it is well known that, compared to highly and moderately differentiated tumors, poorly or undifferentiated tumors and cholangiocarcinoma have a poorer prognosis. In our study, we also confirmed that the 5-year LCSS of poor and undifferentiated tumors, and cholangiocarcinoma was 7.0% and 5.1%, respectively.

In this cohort, we found more patients with poor and undifferentiated grading, more cholangiocarcinoma, and more patients with an advanced stage in the younger groups. Univariate analysis showed that young patients had a better 5-year LCSS in localized, regional, and distant stages compared with the older age group, but this failed to reach statistical significance in multivariable Cox regression models of distant stages (*P* = 0.302). A total of 1795 younger LC patients and 19849 older ones were included in our study, the largest sample size up to now. The fact that this analysis was based on a large sample made our results more convincing. Due to the advanced stage in the younger groups, these patients more frequently underwent major hepatectomy. No significant difference was observed between groups [28]. Young patients have a better survival, which is compensated by better liver function, more aggressive therapy, and faster recovery. Adjuvant chemotherapy is well tolerated in young patients and significantly reduces the risk of tumor recurrence. Although age was not an independent prognostic factor when groups were matched for distant stage, it could be explained by the biological behavior of the tumor. Adjuvant chemotherapy or other therapies have some limitations on prolonging survival in distant stages. Therefore, aggressive treatment is an option for younger LC patients to improve survival.

Although this study is based on a large population and multicenter analysis, there are still limitations. First, its retrospective nature may affect the analysis, due to bias. Second, the information on cancer-specific death may not be precise in the SEER database. Furthermore, the SEER database lacks important information regarding LC predisposing factors (e.g., viral hepatitis, non-alcoholic fatty liver disease, or cirrhosis), cancer treatment (chemotherapy, quality of surgery), as well as alpha-fetoprotein levels. Postoperative morbidity, which was not provided by the SEER database, may have contributed to poor survival in the older age group. Moreover, these potential confounding factors may differ according to age and may not have been adjusted by our analyses, which affects the strength of our results. Importantly, only patients who underwent surgical resection for LC were included in the database, and as such, these patients did not represent LC patients with unresectable tumors. Despite these limitations, our study was based on a large population and multiple centers, and is therefore convincing.

In conclusion, compared to older patients, younger patients with LC (age 45 or below) have a higher LCSS after surgery despite the poorer biological behavior of their carcinomas.

## MATERIALS AND METHODS

### Patients

The SEER Cancer Statistics Review (http://seer.cancer.gov/data/citation.html), a report on the most recent cancer incidence, mortality, survival, prevalence, and lifetime risk statistics, is published annually by the Data Analysis and Interpretation Branch of the National Cancer Institute, (Bethesda, MD, USA). The current SEER database consists of 17 population-based cancer registries that represent approximately 26% of the population in the United States. SEER data contain no identifiers and are publicly available for studies of cancer-based epidemiology and survival analysis. The National Cancer Institute's SEER*Stat software (Surveillance Research Program, National Cancer Institute SEER*Stat software, www.seer.cancer.gov/seerstat) (Version 8.1.5) was used to identify patients whose pathologic diagnosis was LC, based on International Classification of Diseases for Oncology (ICD-O) topography codes (C22.0 and C22.1) between 1988 and 2003, for liver and intrahepatic bile duct cancers, respectively. Morphology codes for liver cancer were expanded to include the following histologies: 8170, 8171, 8172, 8173, 8174, 8175, 8160, and 8180 (i.e., NOS, fibrolamellar, scirrhous, spindle cell variant, clear cell type, pleomorphic type HCC, cholangiocarcinoma, and combined hepatocellular and cholangiocarcinoma). Only patients who underwent surgery with an age at diagnosis between 18 and 85 years were included. Patients were excluded if they had incomplete staging, distant metastasis (M1), no evaluation of histological type, or follow up. Age, sex, race, histologic type, stage, tumor grade, tumor size, and liver cancer specific survival (LCSS) was assessed. Adjuvant chemotherapy was not evaluated as the SEER registry does not include this information. The primary endpoint of the study is LCSS, which was calculated from the date of diagnosis to the date of cancer specific death. Deaths were treated as events and deaths from other causes were treated as censored observation.

This study was based on public data from the SEER database; we obtained permission to access research data files with the reference number 11928-Nov2013. There was no use of human subjects or personal identifying information in this study. The study did not require informed consent, and was approved by the Review Board of Nanjing Medical University, Nanjing, China.

### Statistical analysis

The association of age (young and elderly) with clinicopathologic parameters was analyzed by the chi-squared (χ2) test. Continuous variables were analyzed using the Student's *t*-test. Survival curves were generated using Kaplan–Meier estimates; differences between the curves were analyzed by log-rank test. Multivariable Cox regression models were built for analysis of risk factors for survival outcomes. All statistical analyses were performed using the statistical software package SPSS for Windows, version 17 (SPSS Inc., Chicago, IL, USA). Results were considered statistically significant when a two-tailed test of a *p* value of less than 0.05 was achieved.

## SUPPLEMENTARY TABLES


