# Supplementary material for: Impact of age on the survival of patients with liver cancer: an analysis of 27,255 patients in the SEER database
Source: Oncotarget. 2015 Jan 21;6(2):633–41. doi: 10.18632/oncotarget.2719 (PMC4359244; doi:10.18632/oncotarget.2719)
Supplement: Supplementary file 1 [file oncotarget-06-633-s001.pdf]

SUPPLEMENTARY TABLES

Supplementary Table 1A: Characteristics of HCC patients from SEER Database by age

Characteristic	Total	Young Group	Elderly Group	*p* value
	**23547**	**1846**	**21701**	
Years of diagnosis				$P < 0.001$
1988–1993	3730	337	3393	
1994–1999	7264	612	6652	
2000–2003	12553	897	11656	
Sex				$P < 0.001$
Male	17241	1444	15797	
Female	6306	402	5904	
Race				$P < 0.001$
Caucasian	15098	924	14174	
African American	2768	268	2500	
Others*	5681	654	5027	
Pathological grading				$P < 0.001$
High/Moderate	5888	411	5477	
Poor/ undifferentiation	2445	265	2180	
Stage				$P < 0.001$
Localized	8286	581	7705	
Regional	6209	548	5661	
Distant	4428	432	3996	
Tumor size				$P < 0.001$
< 3cm	1872	148	1724	
3–5cm	3781	228	3553	
> 5cm	7003	638	6365	

Supplementary Table 1B: Characteristics of ICC Patients from SEER Database by age

	Total	Young Group	Elderly Group	*p* value
Characteristic	3502	228	3274	
Years of diagnosis				0.936
1988–1993	726	49	677	
1994–1999	1219	80	1139	
2000–2003	1557	99	1458	
Sex				0.746
Male	1956	125	1831	
Female	1546	103	1443	
Race				0.097
Caucasian	2648	172	2476	
African American	279	11	268	
Others*	575	45	530	
Pathological grading				0.198
High/Moderate	627	65	562	
Poor/ undifferentiation	516	42	474	
Stage				$P < 0.001$
Localized	744	34	710	
Regional	814	74	740	
Distant	987	98	889	
Tumor size				0.177
< 3cm	199	19	180	
3–5cm	334	22	312	
> 5cm	558	57	501	

*including other (American Indian/AK Native, Asian/Pacific Islander) and unknowns.

Supplementary Table 2A: Univariate survival analyses of HCC patients according to various clinicopathological variables

Variable	n	5-year LCSS (%)	Log rank χ2 test	*p* value
Years of diagnosis			426.96	$P < 0.001$
1988–1993	3730	4.6%		
1994–1999	7264	7.8%		
2000–2003	12553	11.9%		
Sex			6.14	$P < 0.05$
Male	17241	9.3%		
Female	6306	10.0%		
Age			69.41	$P < 0.001$
≤ 45	1846	15.3%		
> 45	21701	9.0%		
Race			184.28	$P < 0.001$
Caucasian	15098	9.1%		
African American	2768	6.0%		
Others*	5681	12.2%		
Pathological grading			530.71	$P < 0.001$
High/Moderate	5888	18.6%		
Poor/ undifferentiation	2445	7.4%		
Stage			2849.69	$P < 0.001$
Localized	8286	19.2%		
Regional	6209	5.9%		
Distant	4428	1.6%		
Tumor size			985.25	$P < 0.001$
< 3cm	1872	33.1%		
3–5cm	3781	16.4%		
> 5cm	7003	8.1%		

Supplementary Table 2B: Univariate survival analyses of ICC patients according to various clinicopathological variables

Variable	n	5-year LCSS (%)	Log rank χ2 test	*p* value
Years of diagnosis			25.74	$P < 0.001$
1988–1993	726	3.2%		
1994–1999	1219	4.8%		
2000–2003	1557	6.2%		
Sex			1.73	0.188
Male	1956	4.4%		
Female	1546	6.0%		
Age			16.88	$P < 0.001$
≤ 45	228	8.9%		
> 45	3274	4.8%		
Race			3.81	0.149
Caucasian	2648	5.2%		
African American	279	5.8%		
Others*	575	4.0%		
Pathological grading			530.71	$P < 0.001$
High/Moderate	5888	18.6%		
Poor/ undifferentiation	2445	7.4%		
Stage			255.137	$P < 0.001$
Localized	744	11.6%		
Regional	814	4.2%		
Distant	987	1.0%		
Tumor size			3.779	0.151
< 3cm	199	12.9%		
3–5cm	334	8.5%		
> 5cm	558	9.4%		

*including other (American Indian/AK Native, Asian/Pacific Islander) and unknowns.

Supplementary Table 3A: Multivariate Cox model analyses of prognostic factors of HCC

Variable	Hazard Ratio	95%CI	*p* value
Years of diagnosis			$P < 0.001$
1988–1993	1		
1994–1999	1.075	0.961–1.204	
2000–2003	0.914	0.822–1.015	
Sex			$P < 0.05$
Male	1		
Female	0.912	0.850–0.978	
Age			$P < 0.001$
≤ 45	1		
> 45	1.301	1.162–1.456	
Race			$P < 0.001$
Caucasian	1		
African American	1.145	1.033–1.268	
Others*	0.846	0.786–0.911	
Pathological grading			$P < 0.001$
High/Moderate	1		
Poor/undifferentiation	1.460	1.362–1.564	
Stage			$P < 0.001$
Localized	1		
Regional	1.520	1.420–1.627	
Distant	2.529	2.300–2.780	
Tumor size			$P < 0.001$
< 3cm	1		
3–5cm	1.568	1.419–1.733	
> 5cm	2.050	1.865–2.253	

Supplementary Table 3B: Multivariate Cox model analyses of prognostic factors of ICC

Variable	Hazard Ratio	95%CI	*p* value
Years of diagnosis			0.356
1988–1993	1		
1994–1999	1.008	0.836–1.216	
2000–2003	0.916	0.763–1.101	
Age			$P < 0.05$
≤ 45	1		
> 45	1.226	0.992–1.514	
Pathological grading			$P < 0.001$
High/Moderate	1		
Poor/undifferentiation	1.291	1.136–1.467	
Stage			$P < 0.001$
Localized	1		
Regional	1.670	1.416–1.969	
Distant	2.566	2.170–3.035	

*including other (American Indian/AK Native, Asian/Pacific Islander) and unknowns.
